# Sex determination mode does not affect body or genital development of the central bearded dragon (*Pogona vitticeps*)

**DOI:** 10.1186/s13227-017-0087-5

**Published:** 2017-12-04

**Authors:** Sarah L. Whiteley, Clare E. Holleley, Wendy A. Ruscoe, Meghan Castelli, Darryl L. Whitehead, Juan Lei, Arthur Georges, Vera Weisbecker

**Affiliations:** 1 School of Biological Sciences, University of Queensland, Brisbane, QLD Australia; 2 Australian National Wildlife Collection, National Research Collections Australia, CSIRO, Canberra, ACT Australia; 3 Institute for Applied Ecology, University of Canberra, Canberra, ACT Australia; 4 School of Biomedical Science, University of Queensland, Brisbane, QLD Australia

**Keywords:** Sex reversal, Genitalia, Embryonic development, Staging table, Squamates

## Abstract

**Background:**

The development of male- or female-specific phenotypes in squamates is typically controlled by either temperature-dependent sex determination (TSD) or chromosome-based genetic sex determination (GSD). However, while sex determination is a major switch in individual phenotypic development, it is unknownhow evolutionary transitions between GSD and TSD might impact on the evolution of squamate phenotypes, particularly the fast-evolving and diverse genitalia. Here, we take the unique opportunity of studying the impact of both sex determination mechanisms on the embryological development of the central bearded dragon (*Pogona vitticeps*). This is possible because of the transitional sex determination system of this species, in which genetically male individuals reverse sex at high incubation temperatures. This can trigger the evolutionary transition of GSD to TSD in a single generation, making *P. vitticeps* an ideal model organism for comparing the effects of both sex determination processes in the same species.

**Results:**

We conducted four incubation experiments on 265 *P. vitticeps* eggs, covering two temperature regimes (“normal” at 28 °C and “sex reversing” at 36 °C) and the two maternal sexual genotypes (concordant ZW females or sex-reversed ZZ females). From this, we provide the first detailed staging system for the species, with a focus on genital and limb development. This was augmented by a new sex chromosome identification methodology for *P. vitticeps* that is non-destructive to the embryo. We found a strong correlation between embryo age and embryo stage. Aside from faster growth in 36 °C treatments, body and external genital development was entirely unperturbed by temperature, sex reversal or maternal sexual genotype. Unexpectedly, all females developed hemipenes (the genital phenotype of adult male *P. vitticeps*), which regress close to hatching.

**Conclusions:**

The tight correlation between embryo age and embryo stage allows the precise targeting of specific developmental periods in the emerging field of molecular research on *P. vitticeps*. The stability of genital development in all treatments suggests that the two sex-determining mechanisms have little impact on genital evolution, despite their known role in triggering genital development. Hemipenis retention in developing female *P. vitticeps*, together with frequent occurrences of hemipenis-like structures during development in other squamate species, raises the possibility of a bias towards hemipenis formation in the ancestral developmental programme for squamate genitalia.

**Electronic supplementary material:**

The online version of this article (10.1186/s13227-017-0087-5) contains supplementary material, which is available to authorized users.

## Background

One of the most fundamental aspects of any sexually reproducing organism is its phenotypic sex, as this profoundly influences many aspects of its life history and eventual reproductive success [1]. In squamates, sexual development is controlled by a variety of mechanisms resulting from a dynamic evolutionary history [2]. These can be broadly categorised into temperature-dependent sex determination, genetic sex determination [1, 3–7] and systems where genotype and environment interact to determine sex [8, 9]. Temperature-dependent sex determination (TSD)—where sex is determined by incubation temperature during the “thermosensitive period”—occurs in all crocodiles, many turtles, the tuatara, and seems to be the predominant mechanism of sex determination for lizards [10–15]. By contrast, genetically controlled sex determination (where genes on sex chromosomes determine sexual phenotypes; GSD) occurs in snakes and some lizards and turtles [3, 16–20]. The evolutionary history of sex-determining mechanisms (SDMs) is remarkably diverse in squamates when compared with mammals, whose sex chromosomes have a single origin [21]. Squamate sex chromosomes have independently evolved in many lineages, and transitions from TSD to GSD systems can occur within short evolutionary time frames [12, 18, 22–25].

The conserved development of external genitalia (hereafter referred to as genitalia) in squamates is thought to be controlled by hormones secreted after sex determination, a process generally regarded as being unperturbed by squamates’ various SDMs [26–31]. However, as any comparative study of genital development would be phylogenetically confounded, this assumption has not been properly tested. Given that cell-autonomous sex has been demonstrated in birds, and there are instances of intersexuality and gynandromorphism in squamates, it is possible that genital development is influenced by mechanisms other than gonadal hormones, which could be perturbed by different SDMs [32–37].

To investigate the developmental effects of different SDMs, particularly on genital morphology, we used an experimental approach in a unique model system, *Pogona vitticeps*. This species exhibits genotypic sex determination (ZZ/ZW female heterogametic system [38]), but incubation temperatures at or above 32 °C can cause the complete phenotypic feminisation of genetically male (ZZ) individuals [8, 14, 39]. *P. vitticeps* is one of only two reptile species known to exhibit thermally triggered sex reversal in wild populations (the other being the Eastern Three-Lined Skink, *Bassiana duperreyi* [39]). *P. vitticeps* is also the only reptile in which a rapid transition from GSD to TSD has been experimentally triggered through the mating of male and female homogametic individuals [14]. This provides a unique opportunity to examine embryonic development under both chromosomal and temperature influence within the same species.

Our study is the first to characterise and compare the developmental effects of different incubation temperatures on offspring from concordant (ZWf) and sex-reversed (ZZf) mothers in *P. vitticeps*, including the first assessment of developmental patterns associated with temperature-induced sex reversal. For this purpose, we provide a comprehensive embryonic staging table for *P. vitticeps*, with a particular focus on describing the effects of temperature and genetic sex determination on the development of male and female genitalia.

Using a new molecular approach to identify embryonic genotypes, we assess for the first time whether development, particularly of the genitalia, is perturbed by differing SDMs (GSD vs. TSD) or sex reversal in the same species. We also ask whether staging accurately describes gross embryonic development in different incubation regimes. This allows us to provide the first macroevolutionary perspective on how sex determination mechanisms may impact on the phenotype of the body and particularly genitalia of squamates.

## Methods

### Breeding and incubation treatments

**Fig. 1:**
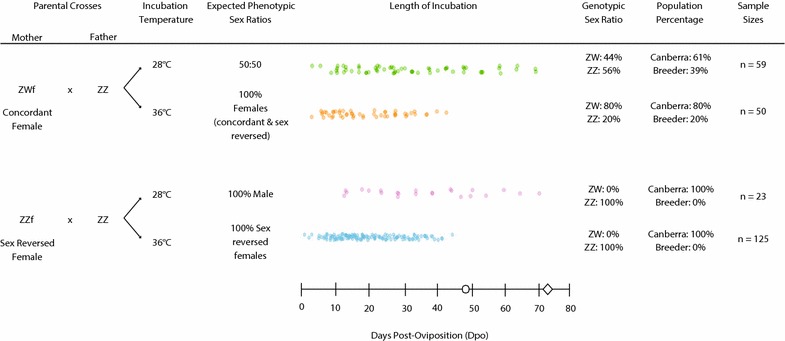
Experimental design encompassing all procedures used in this study. Circle denotes approximate day of hatching for eggs incubated at 36 °C (46.7 ± 1.6 SD) and diamond for eggs incubated at 28 °C (73 ± 3.5 SD) based on estimates from Holleley et al. [14]

To assess developmental differences between GSD and TSD breeding lines of *P. vitticeps*, we crossed ZZ males with ZWf (concordant) and ZZf (sex-reversed) females. Eggs were collected upon laying and allocated into four experimental treatments to produce all offspring phenotypes resulting from combinations of high and low temperatures (28, 36 °C) and maternal genotypes (ZZ, ZW; Fig. 1). The 28ZW treatment provided a baseline for normal development, as sex under these conditions is genetically determined (ZZ males, ZW females). The 36ZW treatment is expected to yield approximately 50:50 concordant (ZWf) and sex-reversed (ZZf) females, making it possible to compare concordant and sex-reversed development at the same temperature. The 36ZZ treatment documented the development of sex-reversed females from sex-reversed mothers, while the 28ZZ treatment yielded concordant males from sex-reversed mothers.

During the 2015–2016 breeding seasons, a total of 254 eggs were incubated and sampled. Of these, 221 eggs were obtained from the University of Canberra’s (UC) captive breeding colony (1–3 generations from animals sourced from a wild population in northern New South Wales/South West Queensland). An additional 33 eggs were sourced from the commercial pet trade and incubated at the University of Queensland (10 sampled in the 36ZW treatment and 23 in the 28ZW treatment). All specimens were staged and photographed using a Dino-Lite Edge digital microscope after formalin preservation.

Due to issues with formalin preservation, early developmental stages (prior to stage 4), including stage at oviposition, were not captured during this initial sampling effort. To obtain these stages, 8 eggs were sampled on the day of oviposition (four different mothers; two ZZ and two ZW) and three stage 2–4 embryos (single ZZ mother, incubated at 36 °C) were obtained from UC’s colony during the 2017 breeding season. All specimens were staged and photographed using a Leica Wild MZ8 dissection microscope prior to formalin preservation.

All eggs were incubated in damp vermiculite (four parts vermiculite to five parts water by weight) in constant temperature incubators with high humidity and minimal temperature fluctuations outside of the set range (± 1 °C range, excluding fluctuations arising from examination of the eggs). Eggs from all mothers (*n* = 17 breeding females) were allocated to the four treatments and sampled across development (Fig. 1). Initially, the entirety of development was surveyed by randomly assigning eggs to sampling days to establish baseline data on developmental timing (every 3 days at 36 °C and every 5 days at 28 °C). This survey identified critical periods of development, which required higher-resolution sampling to determine the timing and order of developmental events (Fig. 1). Sampling intensity was greater for the 36ZZ treatment (sex-reversed offspring of sex-reversed mothers) to ensure that any morphological diversity associated with temperature-induced sex reversal was adequately described.

Embryos and intact yolks were dissected from the egg, and all embryos sampled after the first third of the incubation period were humanely euthanised by intracranial injection of 100 µl of sodium pentobarbitone (60 mg/ml; [40]). Embryos were kept in 10% neutral-buffered formalin fixative for a minimum of 24 h (no more than 72 h), then rinsed in water and stored in 70% ethanol. After ethanol preservation, which stabilises the yolk and embryo for handling, all embryos and yolks were weighed separately for analysis of growth and yolk absorption rates. Ethanol dehydrates tissues; thus, the embryo and yolk weights in this study may slightly underestimate the weight prior to preservation. However, because all specimens were subjected to the same preservation conditions, this approach is unlikely to have introduced systematic bias in our data and is suitable for a general assessment of growth patterns.

### Molecular sex identification in embryonic samples

We developed a novel approach that is non-destructive to the embryo for molecular sex identification of embryonic specimens. Embryonic blood from the inside of the eggshell was swabbed onto a FTA^®^ Elute Micro Card (Whatman) immediately after egg dissection. DNA was extracted following the manufacturer’s instructions with a protocol adapted for automated high-throughput analysis on the Eppendorf EPmotion 5075 liquid-handling platform. Specifically, in a 96-well plate, 3mmdiameter FTA card punches were individually washed in water, boiled for 30 min (100 °C) in a total volume of 100 µl water and then vortexed for 2 min to release DNA from the card. To concentrate the DNA, samples were incubated in a heat block at 40–50 °C for approximately 2 h, until the elution volume was reduced by 50% via evaporation. To confirm that sufficient DNA was extracted from embryonic blood collected on FTA cards, DNA concentration was quantified for a subset of samples (*n* = 92) both prior to and after controlled sample evaporation using a NanoDrop 1000 spectrophotometer (Thermo Scientific). The DNA concentration of embryonic samples was compared to the DNA concentration from FTA^®^ card extractions of adult *P. vitticeps* blood samples (*n* = 30).

We then conducted a PCR-based test, which is diagnostic for the presence of the W chromosome. PCR conditions followed Holleley et al. [14]; however, due to the likelihood of low DNA concentrations from embryonic material, we increased the volume of DNA added to PCRs (3 µl per reaction; approximately 65 ng DNA per PCR). Using primers H2 and F [41], two bands amplify in ZW individuals, whereas a single control band amplifies in ZZ individuals. Animals showing genotype–phenotype discordance were classified as sex-reversed. To test the level of confidence with which we could assign a genotype to embryos sampled with the non-destructive approach, we compared genotype results from blood taken directly from the embryonic heart to the non-destructively sampled eggshell swabs in a subset of specimens (*n* = 23). All PCR tests were run in triplicate, and maternal contamination was investigated by checking phenotypically male embryos (obligate ZZ genotype) for the presence of W chromosome contamination.

### Developmental staging

Staging was based on Sanger et al. [40] staging system for *Anolis* spp, but also included characters from Wise et al. [13] staging system for the leopard gecko (*Eublepharis macularis*). Stages based on traits not present in *P. vitticeps* (digital pad, toe lamellae), or that were not diagnostic for a given stage in *P. vitticeps* (scale anlagen, first full scales, pigmentation), were renamed. In addition, we developed novel staging criteria that described genital development. Specimens obtained from the commercially bred line (*n* = 33) were not used to establish pigmentation development, as pigmentation patterning obviously differed to that of the wild-derived breeding colony (likely due to selective breeding for colour variation in the pet trade).

To quantify how well age as a function of stage explained embryo growth (defined as embryo weight over age), and whether there were differences between treatments, models were fit to a linear equation (Stage = a + b * Age) with treatment as fixed effect, using the *nls* function in R version 3.2.2. Subsequently, we investigated whether the relationship between age and stage was different between temperature and maternal type (sex-reversed ZZ mother or concordant ZW mother) treatments using the *nlme* function of the *nlme* package. A random maternal effect was incorporated into the model to account for maternal effects as clutches from 17 different mothers were distributed across the study. Our data set was too small to incorporate maternal types (ZZ/ZW mothers) across both temperature treatments while including the effects of having 17 mothers in total as well, so we first compared the growth of ZZ versus ZW treatments within temperatures. If these regressions were not significantly different in slope and intercept, we pooled them and compared these pooled data between temperatures.

### Embryo growth and yolk consumption

Embryo growth was estimated using the relationship between weight (g) over time (age, days post-oviposition), with an exponential curve fitted for each treatment using the *nls* function in the *nlme* package for R version 3.2.2, with treatment as fixed effect and mother as random effect. As with the age versus stage comparison, we first compared ZZ/ZW treatments within temperatures, and if no significant differences were found, we pooled treatments and compared between temperatures.

For a visual assessment of the relationship between embryo growth, embryo stages, and yolk consumption in the four treatments, we also plotted log embryo weight and log yolk weight against age (days post-oviposition, dpo).

## Results

### Staging and age prediction by stage

**Fig. 2:**
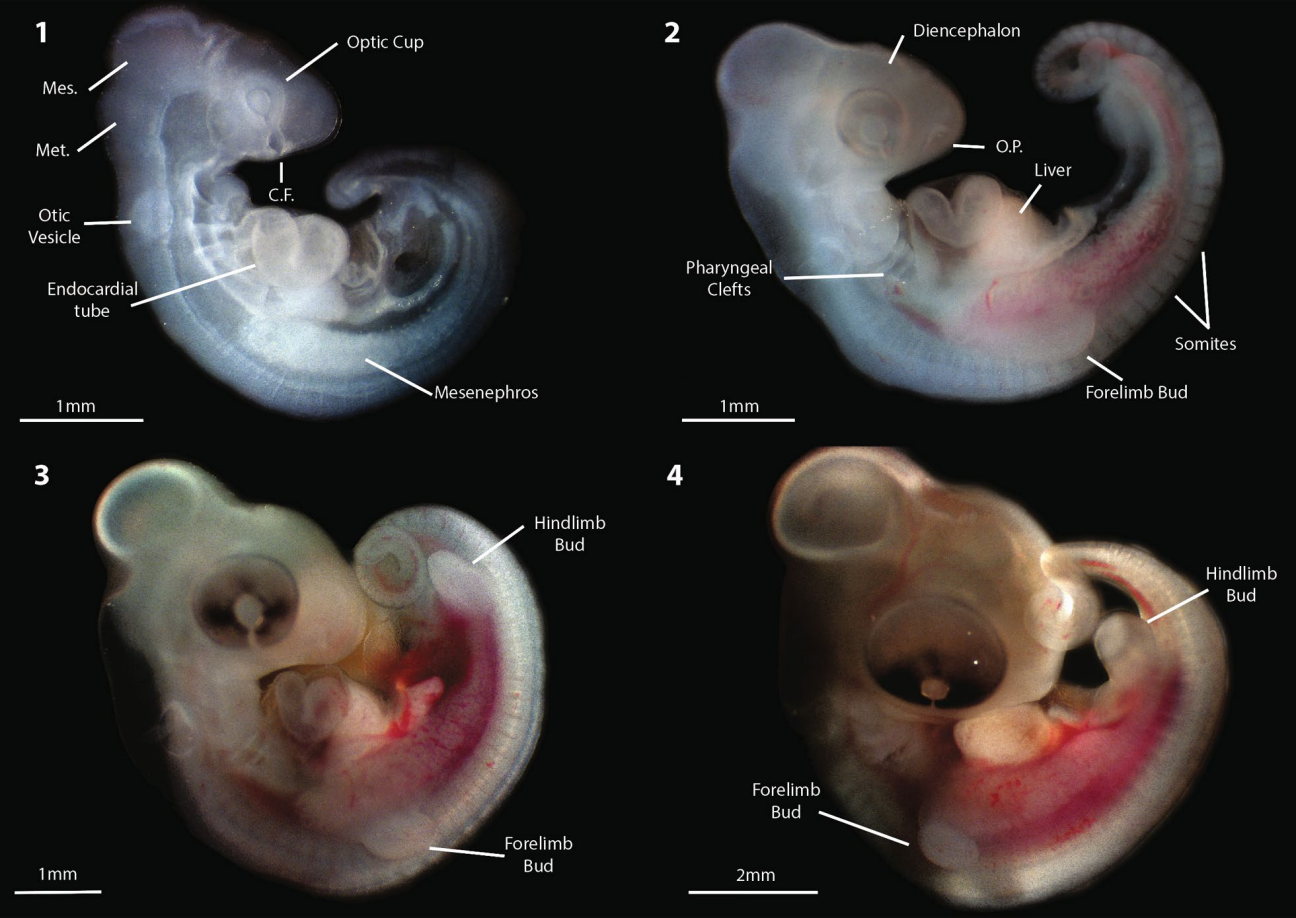
Early developmental stages for *Pogona vitticeps*; stages 1 (day of lay) to 4 (see Table 1). All specimens were photographed prior to formalin preservation. *Mes.* Mesencephalon, *Met.* Metencephalon, *C.F.* choroid fissure, *O.P.* olfactory placodes

**Fig. 3:**
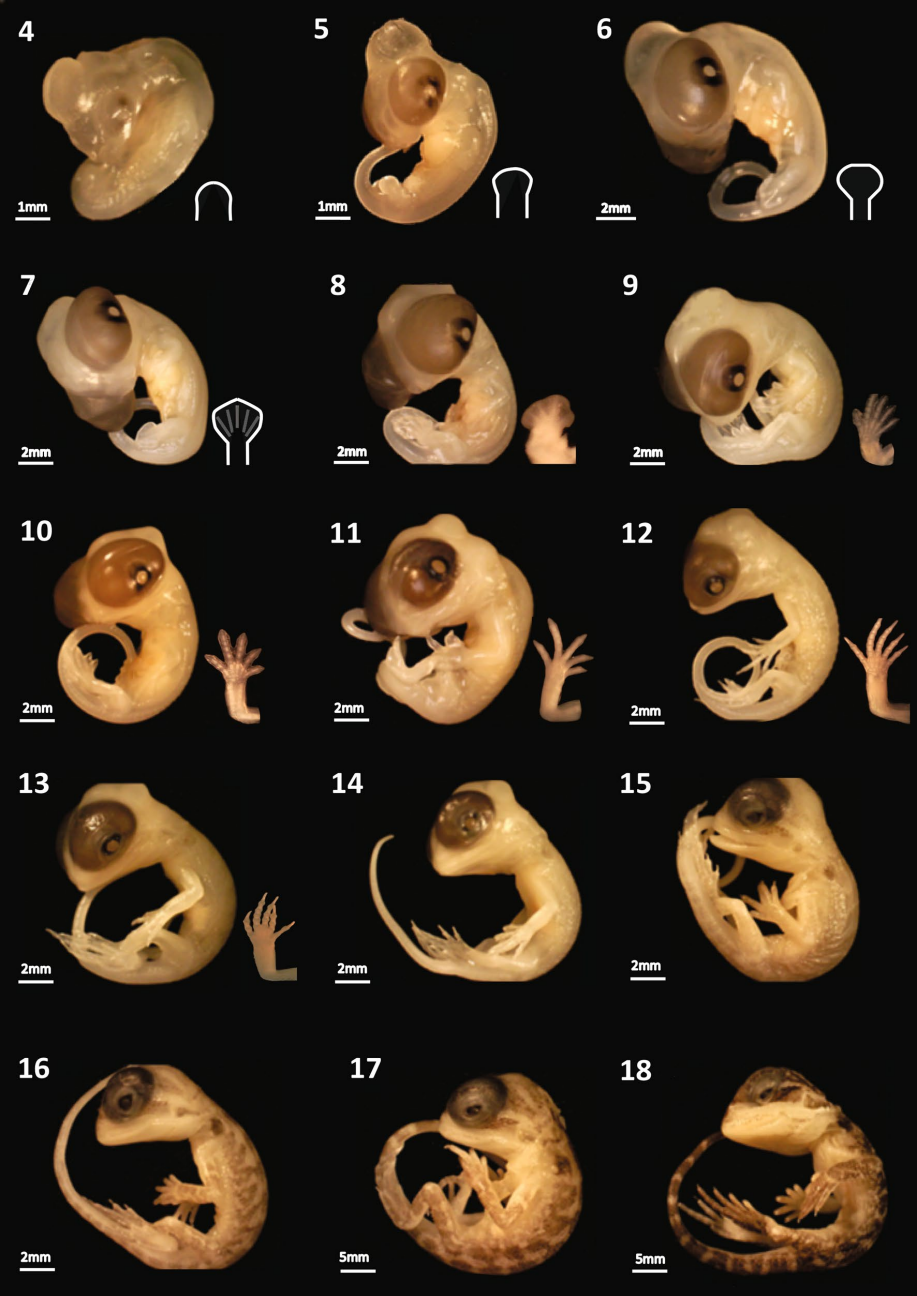
Developmental staging series for *Pogona vitticeps,* depicting stages 5–18 observed across all experimental treatments (see Table 1). All specimens were photographed after formalin preservation

For the staging table and relevant morphology, see Figs. 2, 3; Table 1 and videos of live early-stage embryos (see Additional files 1: Video S1, 2: Video S2 and 3: Video S3). For specimen staging, ageing, genotyping and weights, see Additional file 4: Table S1. Embryos at day of oviposition were less developed than the earliest stages at lay described for *Anolis* spp. and *E. macularius* [13, 40]. While embryos can develop in the oviducts before oviposition, introducing variation in stage of development at lay, we found that eggs were consistently laid at stage 1 (late pre-limb bud; Fig. 1).

**Table 1:** Developmental staging table for *P. vitticeps* based on Sanger et al. [40] staging system for *Anolis* lizards

Stage	Description
1. Late pre-limb bud	*Limbs*: Not yet present *Cranial*: The neural tube is open, extending to the cranial margin of the metencephalon. The mesencephalon forms a conspicuous bulge, with little definition between it and the metencephalon *Eye*: Optic cups are visible as round transparent protuberances with open choroid fissures *Thorax*: The heart is present as a single, folded transparent endocardial tube enclosed within a membrane. Four pharyngeal clefts are open. The mesonephros is present, as are the otic vesicles *Somites*: Approximately 35 somites are present with no obvious tail bud
2. Forelimb bud	*Limbs*: The forelimb buds are present. The hindlimbs are beginning to form as slight thickening of the mesoderm close to the caudal terminus of the embryo *Cranial*: The neural tube remains open, extending to the caudal margin of the mesencephalon. Early meso-metencephalic constriction is apparent, and the metencephalon and mesencephalon become more distinct. The diencephalon becomes more prominent. The olfactory placodes are visible *Eye*: The choroid fissure is obviously narrower, while the lens and optic cups become more defined and very faintly pigmented *Thorax*: Three pharyngeal clefts are open. The mesonephros is larger, extending behind the presumptive liver, which has also increased in size from the previous stage. The margins of the otic vesicles become more defined. The tail bud begins to form *Somites*: Have increased in number from the previous stage (approximately 46), extending past the thickening hindlimb mesoderm, but terminating before the tail bud
3. Hindlimb bud	*Limbs*: Both the fore and hindlimbs are becoming increasingly defined and are beginning to separate from the mesoderm. All limbs are approximately the same size *Cranial*: Meso- and metencephalic constriction increases, giving the mesencephalon a more rounded appearance, and causing the metencephalon to appear more protuberant. The diencephalon becomes more apparent *Eye*: The choroid fissure remains open, and the lens and optic cup become more distinct as pigmentation darkens on the lens *Thorax*: Two pharyngeal clefts are open. All organs increase in size. The mesonephros extends higher in the body cavity. The endocardial tube (presumptive heart) constricts. The tail lengthens and begins to curl *Somites*: Continue to increase in number as they are present past the hindlimb bud and into the developing tail (approximately 48)
4. Early limb bud	*Limbs*: Hind and forelimb buds are well defined and approximately the same size *Cranial*: The margins of the mesencephalon are well defined, creating an obvious division between it and the metencephalon. The diencephalon increases in size. The neural tube narrows *Eye*: Little change from previous stage *Thorax*: All organs, aside from the embryonic kidney, are developing outside of the body cavity, but are beginning to become more enclosed within developing mesoderm
5. Late limb bud	*Limbs*: Both the hind and forelimb buds have increased in size and begin to show a slight pinching between the length of the limb and the developing hand *Cranial*: The mesencephalon continues to develop as a large, translucent protuberance. The metencephalon reduces, while the diencephalon continues to increase in size *Eye*: The eyes have become much larger and protuberant, taking on a uniform light brown colouration with diffuse black pigmentation surrounding the developing pupil *Thorax*: The heart is internalised, while the intestines remain herniated *Genitalia*: The cloaca begins to form as a small indentation between the hindlimb buds
6. Paddle-shaped limb bud	*Limbs*: Both the hind and forelimbs have a distinct paddle, or spade-like shape, but no delineated phalanges *Cranial*: The mesencephalon becomes slightly less protuberant and translucent, while the diencephalon becomes less translucent and moves towards the mesencephalon *Eye*: The eyes continue to increase in size, while the black pigmentation around the pupil becomes less diffuse *Thorax*: The intestines are almost completely enclosed within the body cavity *Genitalia*: The cloaca continues to become more defined as very small genital swellings begin to form on either side of the cloacal opening, between the hindlimbs
7. Digital plate	*Limbs*: Both the hind and forelimbs have become obviously proximodistally segmented, increase in width and become slightly pointed at the apex. Faint digit condensations are visible *Cranial*: The mesencephalon reduces further and becomes less translucent. There is also some definition of the presumptive paired brain swellings as the diencephalon continues to move towards the mesencephalon *Eye*: The eyes become more protuberant and the eyelid begins to form as a thin, translucent covering of skin around the ventral margin of the eye *Thorax*: All organs have become completely internalised *Genitalia*: The genital swellings increase in size and the anterior and posterior cloacal lips start to develop
8. Digital condensations	*Limbs*: The phalangeal bones have condensed, the interdigital webbing becomes slightly reduced, and the limb joints are more distinct *Cranial*: The mesencephalic lobes become more obviously delineated *Eye*: The overall appearance of the eye remains unchanged from the previous stage, but the eyelids continue to envelop more of the eye *Genitalia*: The genital swellings increase in size and start to take on a slightly club-like shape. The anterior and posterior cloacal lips continue to become more defined
9. Early digital web reduction	*Limbs*: The interdigital webbing continues to reduce so that the distal tips are freed, while the elbow joint becomes more distinct, making the limbs flex at approximately 90 degrees *Cranial*: Four mesencephalic lobes have become delineated, and the pineal eye is visible. The two posterior lobes are slightly more protuberant than the two anterior lobes *Eye*: The eyelid now covers approximately three-quarters of the eye *Scales*: Epidermal papillae first become visible, particularly along the dorsal surface *Genitalia*: The genitalia continue to grow and develop an increasingly club-like shape, and the cloacal lips continue to thicken
10. Digital webbing partially reduced	*Limbs*: The digital webbing continues to reduce to approximately half the length of the phalanges *Scales*: Epidermal papillae develop along the dorsal surface and margin of the presumptive beard. The epaulettes also start to form *Genitalia*: The genitalia now have a club-shaped appearance, but are not yet bilobed
11. Digital webbing completely reduced	*Limbs*: The phalanges are no longer joined by any interdigital webbing, and there is a slight pinching at the distal tips of each phalange *Cranial*: The posterior mesencephalic bulges become more protuberant *Eye*: The eyelid thickens, creating an almond-like shape around the eye. The pigmentation darkens, and the black pigmentation condenses around the pupil *Scales*: The epidermal papillae become more prominent along the dorsal surface and margins of the presumptive beard. Very faint pigmentation is visible on the developing epaulettes *Genitalia*: Both sexes now exhibit bilobed hemipenes
12. Eyelid margin (digital pad)	*Limbs*: The phalanges become more elongated with some joint definition and increased pinching at the distal tips, but the claws remain transparent *Eye*: They eyelid now covers the eye up to around the margins of the black pigmentation around the pupil *Scales*: Scale anlagen are present along the margins of beard and side of body, and dorsal surface. Some epidermal papillae develop on the dorsal surface along with faint, scattered pigmentation on the developing epaulettes *Genitalia*: Bilobed hemipenes continue to develop in both sexes
13. Dorsal patterning (toe lamellae)	*Limbs*: The claws are well defined and are no longer transparent. All limb joints become well defined *Eye*: The eyelid surrounds the pupil and beings to thicken at its anterior margins, but remains transparent, underneath which the eyes’ darkening pigmentation is still visible *Scales*: Epidermal papillae are now evident across the entire body. Scale anlagen increase in number and become more defined along the beard margins, sides of body and dorsal surface. Very faint, scattered melanophores appear on the developing epaulettes, extending caudally from the margins of the eye and ear hole. Patterning develops along the dorsal surface and the pigmentation slightly darkens *Genitalia*: Bilobed hemipenes continue to develop; however, in some females they begin to reduce in length but retain their bilobed appearance
14. Mesencephalic bulge reduction (scale anlagen)	*Limbs*: The limbs no longer change in shape *Cranial*: The mesencephalic bulges begin to reduce, and the area of the presumptive parietal bone becomes less translucent *Eye*: The anterior margins of the eyelid continue to thicken *Scales and Pigmentation*: Scale anlagen now cover most of the body, and light pigmentation appears on limbs, tail and cranium, while the dorsal patterning continues to slightly darken *Genitalia*: The hemipenes continue to regress in females and remain unchanged in males
15. Full dorsal scales (first full scales)	*Cranial*: The mesencephalic lobes continue to reduce so that the head has an even dome shape in profile. The presumptive parietal bones continue to develop, further reducing the transparency of the skull *Eye*: The eyelids’ anterior margins thicken further, more closely enclosing the pupil, but remains transparent. The whole eye darkens so there is no delineation between pigmentation around the pupil and the rest of the eye *Scales and Pigmentation*: Pigmentation has darkened considerably from pervious stage, and distinct patterns cover the entire dorsal surface. The claws also darken. Overlapping scales cover most of the dorsal surface, including the tail and limbs. Presumptive spines develop along the beard and sides of body *Genitalia*: In females, the genitalia continue to regress so that either the bilobed appearance is retained, or has disappeared so that the genitalia resemble small, even swellings characteristic of hemiclitores
16. Fully developed scales	*Cranial*: The parietal bone continues to develop so that the mesencephalic lobes are less visible *Eye*: The eyelids continue to thicken over the whole eye and have a well-defined almond shape around the pupil *Scales and Pigmentation*: Fully developed scales are now common across the body, particularly along the beard and dorsal surface. Scales begin to become more prominent on the phalanges and eyelids. Pigmentation continues to darken over the whole body, so that distinct patterns are now obvious *Genitalia*: In females, the genitalia generally continue to regress to hemiclitores, however, there are still some specimens that retain bilobed hemipenes or regressed hemipenes
17. Eye reduction (pigmentation)	*Cranial*: The parietal bone is well developed so that the cranium has a smooth dome-like appearance in profile and the mesencephalic lobes are barely visible *Eye*: The eyes become less protuberant, but the pigmentation and eyelid morphology remains unchanged *Scales and Pigmentation*: Scales continue to develop across the body and are now commonly found to be overlapping. Pigmentation darkens across all dorsal surfaces *Genitalia*: Most female specimens now exhibit either regressed hemipenes or hemiclitores
18. Near-hatching	*Cranial*: The partial bone has formed and is now covered in scales and darkly pigmented. The pineal eye is still visible *Eye*: The eyes are significantly less protuberant, but there is still some dark pigmentation visible under the eyelid *Scales and Pigmentation*: The scales are almost completely developed, and the pigmentation patterns look much like those seen in hatchlings *Genitalia*: The genitalia have completely regressed in females so that only the genital ridge is present within the vent. Males possess large bilobed hemipenes

More detail is provided for morphological traits that are not described for *Anolis*, as well as a general characterisation of genital development. Where applicable, stages that were named after diagnostic characters in *Anolis* that are not present in *P. vitticeps*, were renamed with the original name in brackets

Staging is easiest and most accurate early in development when organogenesis and limb development events are more discrete and becomes more difficult and less accurate as the embryos approach hatching because the morphological changes become less distinct. Often *P. vitticeps* embryos showed a combination of traits across two stages, and so were denoted as 0.5 of a stage. Generally, development progressed similarly to *Anolis* (the species described in the original system upon which we based ours), with exception of the far earlier onset and development of pigmentation, and more rapid development of the eyelid in *P. vitticeps*. Early in development (stages 1–3), the somites extend beyond the developing hindlimb towards the tail bud, increasing their number, whereas in *Anolis* the somites do not extend past the hindlimb.

**Fig. 4:**
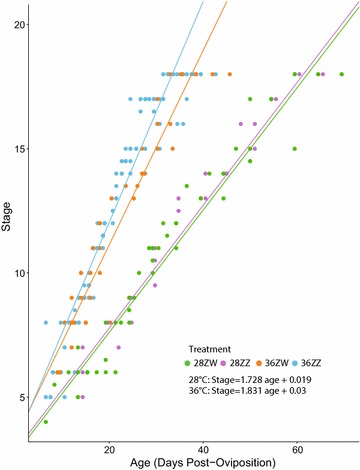
Plot of specimen stages against specimen post-oviposition ages. Growth and stage development are accelerated at high temperatures (36 vs. 28 °C), but are unaffected by the sex chromosome complement of the mother (ZZ vs. ZW)

For each of the treatments, stage as a function of age explained embryo growth very well (Fig. 4; Table 2). Incubation temperature and sex reversal did not influence the order of development of any phenotype. Slopes and intercepts of ZZ and ZW age versus stage fits were not significantly different, although a relatively low *p* value (0.09) of the slope comparison suggests a tendency of 36ZZ specimens to go through later stages slightly more quickly (Fig. 4). A comparison of the slopes between the 36 °C treatments and 28 °C treatments found they were significantly different (Fig. 4; Table 2).

**Table 2:** Summary of age *versus* stage regressions within temperatures and between temperatures, including variance explained by maternal effects

Comparison	df		Variance mat. effect	Value	Std. error	*t* value	*p*
28ZZ-28ZW	68	Intercept	0.601	0.163	0.771	0.211	0.833
	68	Slope	9.1 × 10^−4^	0.0004	0.026	− 0.019	0.985
	68	Residual	0.645				
36ZZ-36ZW	155	Intercept	0.386	− 0.328	0.573	− 0.572	0.568
	155	Slope	0.002	0.057	0.034	1.7	0.091
	155	Residual	0.988				
28 °C–36 °C	232	Intercept					
	232	Slope	0.055	0.124	0.002	9.709	*0.000*
	232	Residual	0.87				

Italics indicate significance at *P* < 0.05

### Sex chromosome genotyping

For details of the embryo genotyping results, refer to Additional file 5: Table S2. As expected, DNA extracted from embryonic material yielded less DNA than an equivalent extraction from adult blood (50.58 ng/µl ± 8.05 SE), both before (13.09 ng/µl ± 1.99 SE) and after evaporative DNA concentration (21.63 ng/µl ± 3.36 SE). However, embryonic DNA yield was sufficient to generate highly reproducible molecular sex identification results. Ninety-nine percentage of samples run in triplicate (*n* = 184 embryonic blood or heart samples) returned concordant results (i.e. all tests ZZ or all tests ZW). Genotype concordance was 100% when comparing embryonic blood with invasively sampled heart blood from the same embryo (*n* = 23). We did not detect any evidence of maternal contamination (presence of W chromosome) in obligate ZZ male embryos from wild-type crosses (*n* = 9). Thus, we are confident that the embryonic blood supply sampled from the interior of an eggshell can be used to assign the genotypic sex of the developing embryo.

### Genital development

**Fig. 5:**
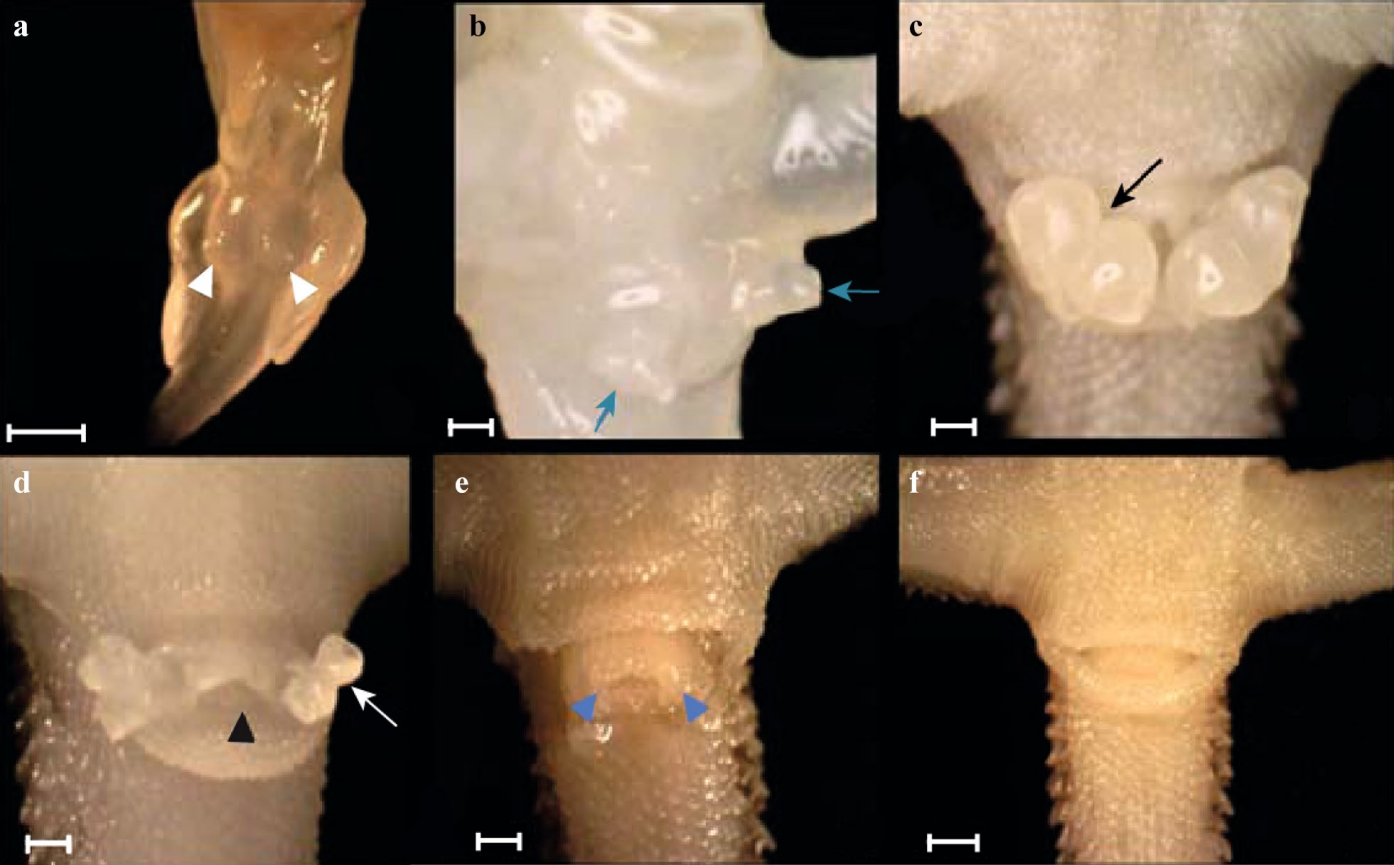
Genital development in female *Pogona vitticeps* embryos. Development progresses from rounded paired swellings between the hindlimbs (**a**, white arrowheads; score 1), club shape (**b**, blue arrows; score 2), bilobed hemipenes (**c**, deepening invaginations create distal bifurcation: black arrow; score 3). The lobes become increasingly accentuated as this invagination deepens (black and white arrows). In females, the hemipenes begin to regress, but maintain their bilobed appearance (**d**, black arrowhead: cloacal opening; score 4). Females eventually possess hemiclitores (**e**, blue arrowheads; score 5) before they too regress completely to the pedicel (**f**; score 6). The anterior and posterior cloacal lips in specimen **e** were removed to expose the hemiclitores. Scale bar = 1 mm

In all sexes (concordant males and females, and sex-reversed females), genital development begins as small paired phallic swellings form on either side of the developing cloaca (between stages 5 and 8 in all treatments, Fig. 5a; score 1 Additional file 4: Table S1). The swellings increase in size until they achieve a club-shaped appearance and are enclosed by distinct anterior and posterior cloacal lips (approximately between stages 9 and 13 in all treatments, Fig. 5b; score 2 Additional file 4: Table S1). This club shape becomes more pronounced as development progresses until the distal tip of each hemipenis is bifurcated, creating the characteristic bilobed appearance of mature hemipenes in all sexes (from approximately stage 11 in all treatments, Fig. 5c; score 3 Additional file 4: Table S1).

Male and female development diverges from stage 11 (73% through development). In males, ongoing development of the hemipenes is characterised by deepening invaginations on the bilobes, which considerably increases their surface area. In all male specimens, the hemipenes were consistently everted; however, in both treatments (28ZW and 28ZZ) a total of fourstage 18 specimens exhibited no everted hemipenes. It was unclear as to whether they were simply folded within the vent as the specimens approached hatching, or were truly absent. In the 28ZW treatment, there were two unexpected phenotypes: one stage 17 (55 dpo) male exhibited reduced hemipenes, while one stage 18 (70 dpo) male exhibited hemiclitores.

In females, hemipenis regression commences at approximately stage 11 with an overall shortening of the hemipenes, but is characterised by retention of the lobes throughout the hemipenis regression process (approximately between stages 11 and 17, Fig. 5d; score 4 Additional file 4: Table S1). The lobes ultimately regress to hemiclitores, which are characterised as two small swellings without bilobes slightly protruding from the margins of the pedicel (from approximately stage 16.5, Fig. 5e; score 5 Additional file 4: Table S1). As embryos approach hatching (stage 18), the hemiclitores reduce completely so that only the pedicel is present at hatching (see Fig. 4f; score 6 in Additional file 4: Table S1). Hence, it is possible to sex hatchlings using traditional methods such as hemipenal eversion [42] and transillumination [43].

### Comparison of embryo growth and yolk consumption

**Fig. 6:**
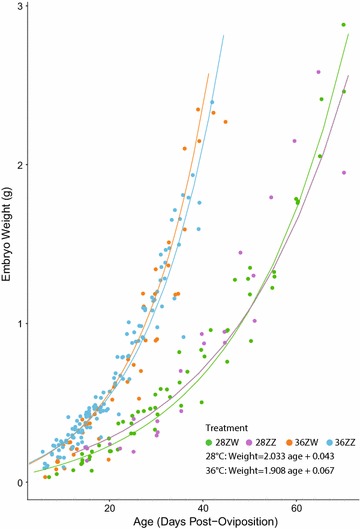
Embryo weight over age follows an exponential curve in all treatments. Growth is faster in the 36 °C treatments than the 28 °C treatments, and growth is unaffected by maternal type (ZZ vs. ZW)

**Fig. 7:**
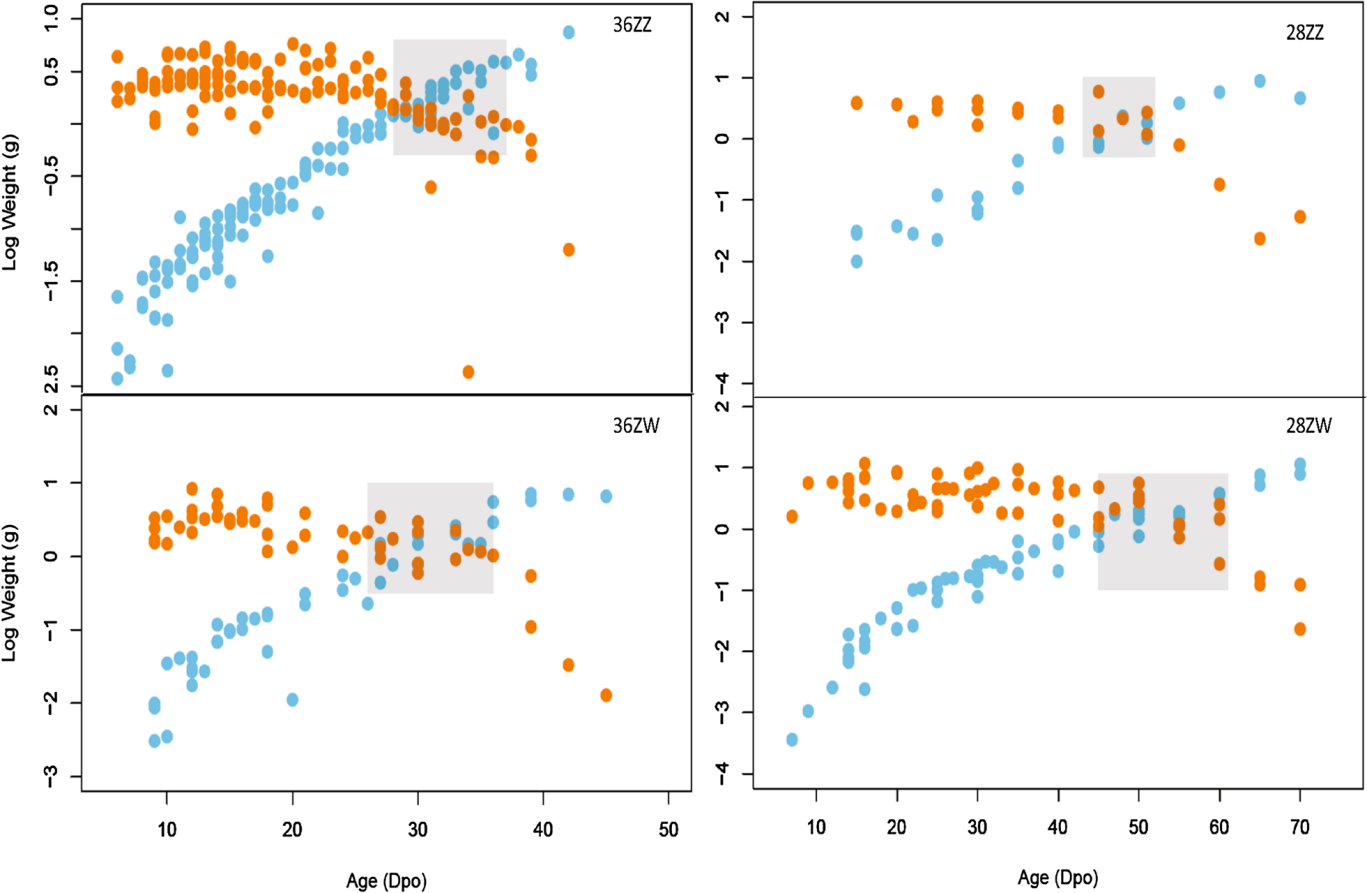
Log embryo (blue) vs. log yolk (orange) weight over time in days post-oviposition (dpo) for each treatment. Shaded rectangle highlights the time at which embryo weight rapidly increases at the expense of yolk weight

Embryo growth follows an exponential curve (Fig. 6), with no differences in slopes between ZZ and ZW offspring within temperature treatments, so we pooled all specimens for each temperature treatment. As predicted, significant differences exist between slopes of the two temperature groups. In all cases, maternal effects were far smaller than the residual variation (Table 3). Early in development, yolk weight is highly variable and not clearly associated with embryo weight (Fig. 7). Later in development, the embryo becomes heavier than the yolk (see shaded areas in Fig. 7). There are no significant differences between offspring from breeder versus wild-obtained mothers in the 36ZW and 28ZW treatments (Additional file 6: Figure S1).

**Table 3:** Summary of growth curve (embryo weight over time) comparisons within temperatures and between temperatures, including variance explained by maternal effects (Var. Mat. Effect)

Comparison	df		Variance mat. effect	Value	Std. error	*t* value	*p*
28ZZ-28ZW	68	Intercept	0.0221	0.15	0.17	0.91	0.37
	68	Slope	1.9 × 10^−15^	− 0.002	0.002	− 1.45	0.15
	68	Residual	0.01				
36ZZ-36ZW	155	Intercept	3.77 × 10^−12^	0.13	0.11	1.18	0.24
	155	Slope	1.37 × 10^−5^	− 0.003	0.004	− 0.79	0.43
	155	Residual	0.014				
28–36 °C	232	Intercept	0.049				
	232	Slope	3.2 × 10^−5^	0.003	0.00003	9.83	*0.000*
	232	Residual	0.014				

Differences in embryo growth rates are explained only by incubation temperature, and not by maternal type

Italics indicate significance at *P* < 0.05

## Discussion

In this study, we provide the first morphological characterisation of external development in *P. vitticeps* under normal and sex-reversing temperatures. Regardless of the sex-determining cue (temperature or sex chromosomes), genital development is a highly conserved process that does not differ between males and females for much of embryonic development. Female development is characterised by the growth, retention, and eventual regression of hemipenes, which are normally characteristic of the male genital phenotype. A review of the literature (Additional file 7: Table S3) reveals that the development of male genitalia in *P. vitticeps* is consistent with the gross morphological processes described for other squamate species. Across temperatures and maternal type, the genital development remains synchronised with the development of other parts of the body, which are also not perturbed in their sequence by either temperature or sex determination mechanism. This observation differs from results in turtles where low temperatures extended the retention of some earlier phenotypes [44]. However, it is possible that similar effects might occur in *P. vitticeps* in particularly cold incubations, which were not included in this study. Regardless, the robustness of genital and phenotypic development to these influences is interesting because in adult sex-reversed females there are differences in fecundity [14], behaviour [45], gene expression [46], and some morphological traits [45]. In contrast, we did not observe any sex-reversal-specific differences in the timing, sequence, or structure of morphological development.

The conserved developmental sequence across temperature treatments and sex determination mechanisms allows an accurate prediction of specimen age from stage for a given temperature in all treatments. Staging is often criticised because there is no standard practice, it usually does not account for the effects of incubation temperature, or differences between field and laboratory raised animals, and often uses small sample sizes [44, 47]. However, these factors had little influence on the accuracy of *P. vitticeps* staging, suggesting that staging remains an ideal method for categorising development. In particular, staging is a powerful method to visually calibrate sampling points in future studies of *P. vitticeps* development, avoiding the need for heavy replication to capture a specific sexual phenotype in this emerging model organism [8, 12, 14, 24, 45, 48, 49].

Our results provide intriguing evidence that sex determination mechanisms (SDMs) do not impact on the formation of *P. vitticeps* genitalia. This suggests that the molecular underpinnings of genital formation through hormonal signalling and dosage from the gonads after sex determination follow the same pattern regardless of whether sex is genetically or temperature-determined [26, 50–52]. This lack of connection between SDMs and genital formation also suggests that the evolution of genital development and SDMs are not closely linked based on current evidence (Additional file 6: Figure S1). However, this needs further investigation across squamates with different SDMs as well as other dual-SDM systems [53, 54].

A robust developmental programme of genital development is not unexpected, as mating success depends on the proper formation of genitalia [26]. However, genitalia are highly diverse within squamates and evolve faster than other phenotypic traits [26, 27, 29]. Based on our results, intraspecific variability or switches in SDM are unlikely to be a source for this diversity; future comparative study of squamate genital phenotypes may provide further insights into the mechanisms driving the evolution of squamate genital morphology.

The extended retention of male traits in female *P. vitticeps* is interesting in an evolutionary context because female genitalia exhibit a far wider range of genital phenotypes than males, but these phenotypes are generally based on the default of a hemipenis form. Female genitalia in squamates vary from structures resembling rudimentary hemipenes to species where females have longer hemipenes and associated musculature than males [33, 35, 50, 55–59]. In *P. vitticeps*, extended developmental hemipenis retention in females and male sex chromosome homogamety suggest that the ancestral programme of genital development may be biased towards hemipenis formation. The acquisition of a developmental pathway for hemipenis regression, which seems to be a secondary occurrence in *P. vitticeps*, may also occur in other species, possibly driven by sexual selection. Although this is speculative, it is consistent with suggestions that the developmental programme governing hemipenis formation is extremely conserved in amniotes [26]. However, limited data exist on female genital development in squamates, and the mechanistic underpinnings of their growth remain poorly understood [51]. This is in contrast to work on males, which is considerably more detailed and addresses the evolutionary and genetic processes governing hemipenis development (Additional file 6: Figure S1). Future studies should consider female development, in particular the developmental processes governing the growth of the genitalia, to improve our understanding of sexual development, particularly in sexually labile species such as *P. vitticeps*.

We observed that *P. vitticeps* eggs were consistently laid at stage 1, which is earlier than described for most other squamates (Fig. 2; Additional file 7: Table S3). *Anolis* were laid at stage 4 (early limb bud), while *E. macularius* were laid at stage 2. A final interesting observation was the variability of yolk weights compared to embryo weight, particularly early in development, across all treatments (Fig. 7). After this phase of large variability, a rapid decrease in yolk beginning from stages 13–18 coincides with the completion of organogenesis (Table 1). This suggests that the majority of yolk consumption occurs when the embryo has a complete body plan and begins to gain weight in preparation for hatching.

## Conclusions

Our investigation clarifies the evolutionary relationship between sex determination and embryological development in *P. vitticeps*, providing an encouraging perspective on the stability of squamate development, even under environmental extremes that might be expected under climate change. Our data suggest that even substantial perturbations at the very beginning of development (+ 8 °C) may not de-stabilise the formation of functional genital phenotypes. However, exposure to feminising temperatures could negatively impact on population viability if sex ratios become skewed [60]. We highlight the need for larger-scale study of female squamate genitalia, particularly with a view to the intriguing possibility that male and female squamate genitalia might represent variations of a hemipenis-like structure. This also raises questions about the main determinants of squamate genital formation, which appears to be unaffected by the sex-determining mechanism. Our staging system and ability to confidently sex embryos using small quantities of embryonic blood will facilitate further molecular based work on this question.

## Additional files

**Additional file 1: Video S1.** Short video of an embryo at day of oviposition with a beating endocardial tube (future heart).

**Additional file 2: Video S2.** Short video of a stage 2 (2 days post-oviposition at 36 °C) with a beating endocardial tube (future heart).

**Additional file 3: Video S3.** Short video of a stage 3 (3 days post-oviposition at 36 °C) with a beating endocardial tube (future heart).

**Additional file 4: Table S1.** Datasheet of all specimens used in this study, including identifier (row names), age (in days post-lay, or DOO to denote specimen was sampled at oviposition), stage, type of specimen (C = from University of Canberra breeding colony, B = breeder), genotype, embryo weight (WTL), yolk weight (WTY), maternal sexual genotype (Mtype), incubation temperature (Temp), genital maturity score (1–6, see Fig. 4 and results for details), identity of mother, number of clutch (some mothers laid several clutches), treatment name.

**Additional file 5: Table S2.** Genotyping results for all embryos used in this study. ID = specimen identifier, DNA concentration before (pre-Concentration) and after (post-Concentration) evaporation-based sample concentration. Genotyping results are the results from each PCR run (a minimum of 3, maximum of 5 runs), including the consensus genotype. Rows 4–33 show data for DNA concentration in post-hatching specimens for comparison with the embryonic concentration data only.

**Additional file 6: Figure S1.** Comparison between populations: Canberra breeding colony sourced from wild animals (blue), and animals sourced from a commercial breeder (orange) for embryo weight (A, C) and yolk weight (B, D) over time (age, days post-oviposition) for the 36ZW (A, B) and 28ZW (C, D) treatments.

**Additional file 7: Table S3.** Overview of literature on developmental staging of reptiles, including what staging methods were used and their associated author/s. The stages are based on each paper’s respective system. Where possible, details regarding the timing of genital development were included. Only papers describing development under normal conditions with no experimental manipulations were included. Any papers that were not written in English with no translation were omitted. It should be noted that often a sexual characteristic was described for the first time, but this was not necessarily the earliest stage of development. Such instances are marked with an asterisk. NA denotes that no sexual characteristics were described, while N denotes that male characteristics were included but female characteristics were excluded, and dpo denotes days post-oviposition. Where possible, stage at oviposition (SAO) is recorded, with an approximation to *P. vitticeps* SAO in brackets. NA denotes that this information was not applicable (e.g. because the species is viviparous). NR denotes that the SAO was not reported.
