# Supplementary figures and images for: Sex determination mode does not affect body or genital development of the central bearded dragon (Pogona vitticeps)
Source: EvoDevo. 2017 Dec 4;8:25. doi: 10.1186/s13227-017-0087-5 (PMC5716226; doi:10.1186/s13227-017-0087-5)

Weight (Grams)

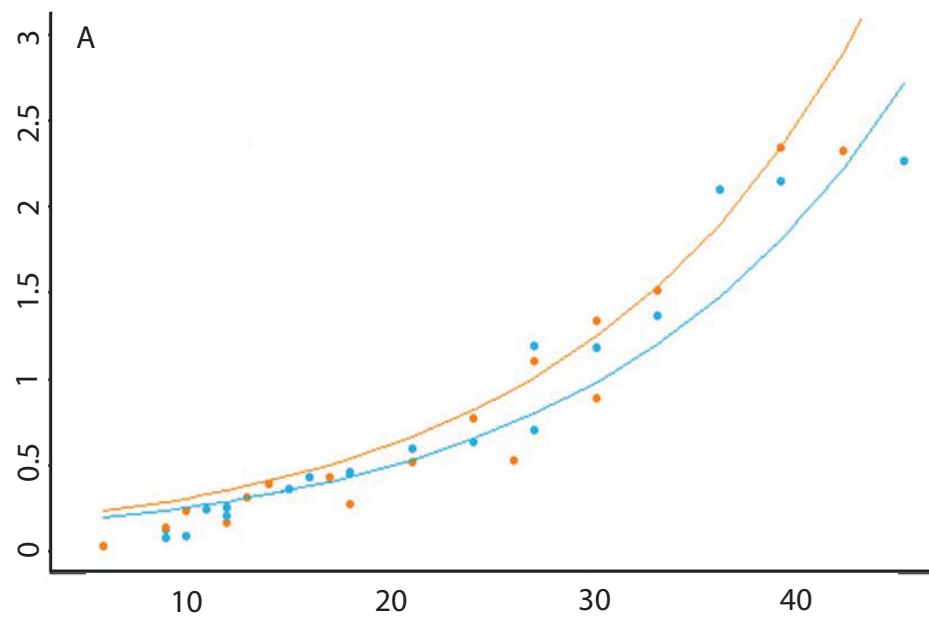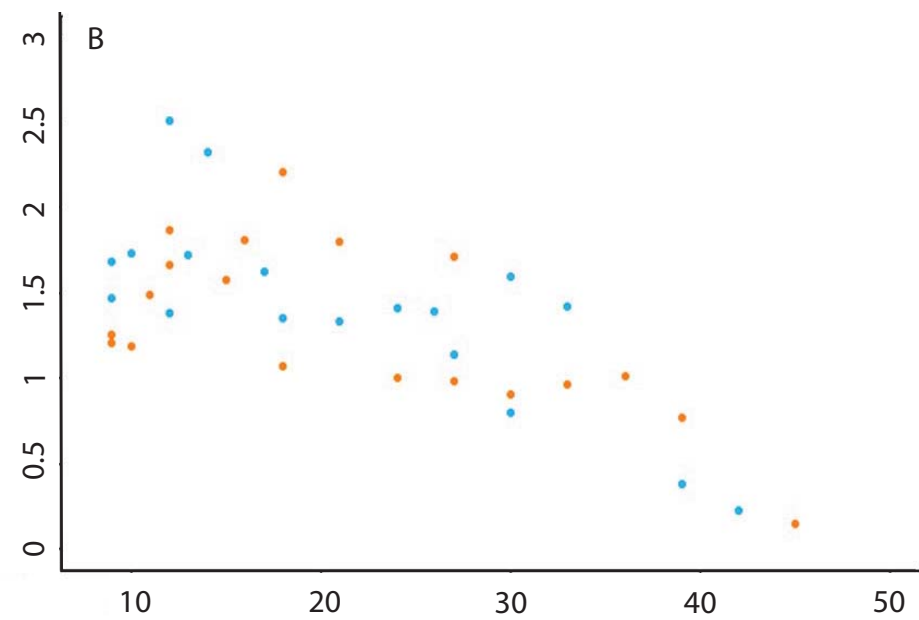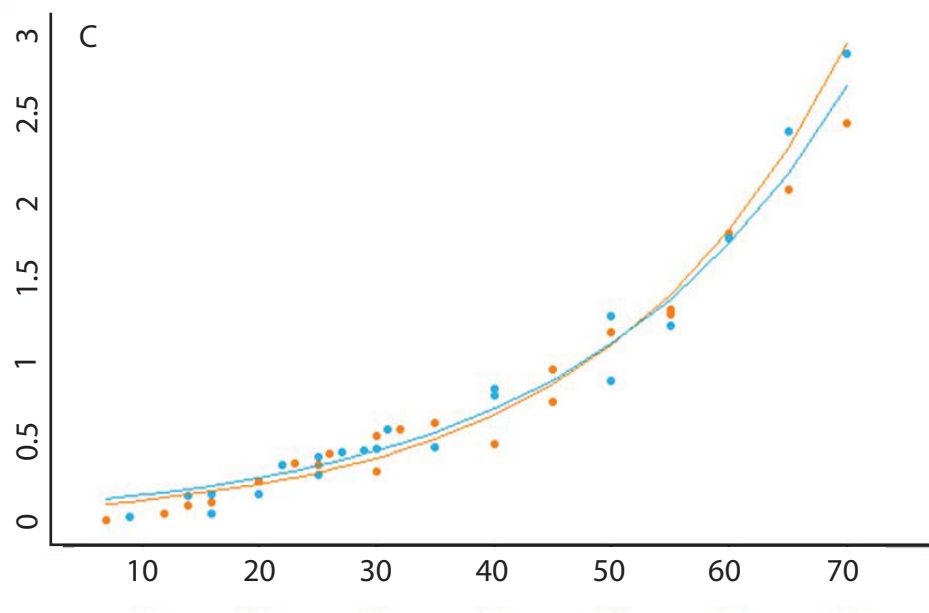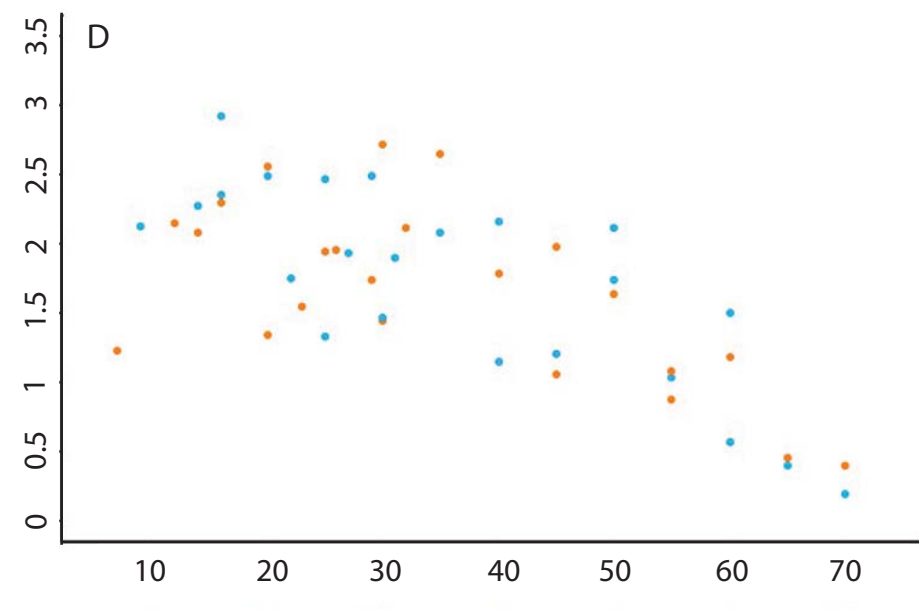

Age (Days Post-Oviposition)

Supplement: Supplementary file 6 — Additional file 6: Figure S1. Comparison between populations: Canberra breeding colony sourced from wild animals (blue), and animals sourced from a commercial breeder (orange) for embryo weight (A, C) and yolk weight (B, D) over time (age, days post-oviposition) for the 36ZW (A, B) and 28ZW (C, D) treatments. [file 13227_2017_87_MOESM6_ESM.pdf]
