# Supplementary material for: Sex determination mode does not affect body or genital development of the central bearded dragon (Pogona vitticeps)
Source: EvoDevo. 2017 Dec 4;8:25. doi: 10.1186/s13227-017-0087-5 (PMC5716226; doi:10.1186/s13227-017-0087-5)
Supplement: Supplementary file 7 — Additional file 7: Table S3. Overview of literature on developmental staging of reptiles, including what staging methods were used and their associated author/s. The stages are based on each paper’s respective system. Where possible, details regarding the timing of genital development were included. Only papers describing development under normal conditions with no experimental manipulations were included. Any papers that were not written in English with no translation were omitted. It should be noted that often a sexual characteristic was described for the first time, but this was not necessarily the earliest stage of development. Such instances are marked with an asterisk. NA denotes that no sexual characteristics were described, while N denotes that male characteristics were included but female characteristics were excluded, and dpo denotes days post-oviposition. Where possible, stage at oviposition (SAO) is recorded, with an approximation to P. vitticeps SAO in brackets. NA denotes that this information was not applicable (e.g. because the species is viviparous). NR denotes that the SAO was not reported. [file 13227_2017_87_MOESM7_ESM.docx]

**Additional file**

**Table** **S3**: Overview of literature on developmental staging of reptiles, including what staging methods were used and their associated author/s. The stages are based on each paper’s respective system. Where possible, any details regarding the timing of genital development was included. Only papers describing development under normal conditions with no experimental manipulations were included. Any papers that were not written in English with no translation were omitted. It should be noted that often a sexual characteristic was described for the first time, but this was not necessarily the earliest stage of development. Such instances are marked with an asterisk. NA denotes no sexual characteristics were described, while N denotes that male characteristics were included but female characteristics were excluded, and dpo denotes days post-oviposition. Where possible, stage at oviposition (SAO) is recorded, with an approximation to *P. vitticeps* SAO in brackets. NA denotes that this information was not applicable (e.g. because the species is viviparous). NR denotes the SAO was not reported.

| Author | Species | Staging Methodology | Stage at Oviposition | Genital  Character | Stage/ dpo | Female data?  (Y/N/NA) |
| --- | --- | --- | --- | --- | --- | --- |
| Dufaure and Hubert 1961 | *Lacerta vivipara* | Developed new system | NR | Early penis development | Stage 31 | Y |
| Zehr 1962 | *Thamnophis sirtalis sirtalis* | Developed new system | NR | Cloacal mound can be seen | Stage 23 | N |
| Hubert and Dufaure 1968 | *Vipera aspis* | Dufaure and Hubert (1961) | NR | Cloacal region develops small swellings | Stage 35 | Y |
| Muthukkaruppan et al. 1970 | *Calotes versicolor* | Dufaure and Hubert (1961) | 27 (2) | The early penis rudiment is present | Stage 32* | Y |
| Lemus et al. 1981 | *Liolaemus tenuis tenuis* | Lemus (1967) | NR | A well-developed phallus is present in both sexes | Stage 37 (21 dpo)* | Y |
| Rosenberg et al. 1989 | *Bradypodion pumilum* | Blanc 1974 | NR | Paired genital protuberances | Stage 36 | N |
| Doddamani, 1994 | *Calotes versicolor* | Muthukkaruppan et al. 1970 | 27 (2) | Genital ridge on either side of the dorsal mesentery | Stage 27 (oviposition) | N |
| Montero et al. 1999 | *Amphisbaena darwini heterozonata* | Developed new system | NR | Eversion of hemipenes | Stage 10* | N |
| Jackson, 2002 | *Naja kaouthia* | Dufaure and Hubert (1961) and Zehr (1962) | NR | NA | NA | NA |
| Neaves et al. 2006 | *Niveoscincus ocellatus* | Dufaure and Hubert (1961) | NA | Hemipenes | Prior to Stage 1 | Y |
| Boughner et al. 2007 | *Python sebae* | Developed new system | 1 (3) | Stage 3* | Hemipenes are visible medial to the limb bud | N |
| Sanger et al. 2008 | *Anolis* (genus) | Developed new system based on limb development | 4 | Stage 10 | Hemipenes obvious in males | N |
| Noro et al. 2009 | *Paroedura pictus* | Sanger et al. (2008) | 0dpo (<1) | Genital buds distinctly bulging at the roots of the hind limbs | 4 dpo | N |
| Wise et al. 2009 | *Eublepharis macularius* | Dufaure and Hubert (1961), Muthukkarruppan et al. (1970), Hamburger and Hamilton (1951), Sanger et al. (2008a); Noro et al. (2009) | 28 (2) | NA | NA | NA |
| Boback et al. 2011 | *Boaedon fuliginosus* | Werneburg (2009), Zehr (1962), Hubert and Dufaure (1968), Jackson (2002), Boughner et al. (2007) | 1 (2) | The hemipenial apparatus first becomes visible | Stage 4 (9-13 dpo) | N |
| Roscito and Rodrigues 2011 | *Nothobachia ablephara* | Werneburg (2009) | NR | Buds of the cranial lip of the cloaca present | Stage 3, (10-12 dpo) | Y |
| Roscito and Rodrigues 2011 | *Calyptommatus sonebrachiatus* | Werneburg (2009) | NR | Hemipenis buds are small, primordia of the cranial lips of the cloaca are present. | Stage 6 (12-14 dpo)* | Y |
| Gregorovicova et al. 2012 | *Varanus indicus* | Dpo, Lemus et al. (1981), Sanger et al. (2008), Noro et al. (2009), Wise et al. (2009) | NR | Cloacal swelling | Stage 8, Dpo 43 | N |
| Khanoon and Evans 2014 | *Naja h. haje* | Zehr (1962), Hubert and Dufaure (1968) | 1 (3) | Distinct cloacal mound and hemipenis analgen | Stage 1 (day of oviposition) | N |
| Leal and Cohn 2014 | *Python regius* | Bougher et al. (2007) and Raynaud (1972) | NA | External genital outgrowth | Stage 1* | N |
| Gredler et al. 2015a | *Anolis carolinensis* | Sanger et al. (2008) | NA | Phallic swelling forms proximally on the posterior-ventral side of the hindlimb bud | Stage 4 | Y |
| Gredler et al. 2015b | *Alligator mississippiensis* | Ferguson 1985 | NA | Genital mesenchyme | Stage 12.5 | N |
| Khanoon 2015 | *Tarentola annularis* | Hamburger and Hamilton (1951), Dufaure and Hubert (1961), Muthukkarruppan et al. (1970), Wise et al. (2009) | 29 (2) | NA | NA | NA |
| Kovtun and Sheverdyukova 2015 | *Natrix natrix* | Zehr (1962) | NA | NA | NA | NA |
| Martinez-Torres et al. 2015 | *Barisia imbricata* | Dufaure and Hubert (1961) | NA | Primordia of hemipenes | Stage 33-34 | Y |
| Py-Daniel et al. 2017 | *Tropidurus torquatus* | Dufaure and Hubert (1961), Sanger et al. (2008), Wise et al. (2009), Werneburg 2009 | 28 (3) | Primordium of external genitalia present | Stage 29 | Y |
| Khanoon and Zahradnicek 2017 | *Psammophis sibilans* | Zehr (1962), Dufaure and Hubert (1968) | 1 (2) | NA | NA | NA |

**References**

Boback, S. M., E. K. Dichter, and H. L. Mistry. 2012. A developmental staging series for the African house snake, *Boaedon (Lamprophis) fuliginosus*. Zoology 115:38-46.

Boughner, J. C., M. Buchtová, K. Fu, V. Diewert, B. Hallgrímsson, and J. M. Richman. 2007. Embryonic development of *Python sebae* – I: Staging criteria and macroscopic skeletal morphogenesis of the head and limbs. Zoology 110:212-230.

Doddamani, L. S. 1994. Histoenzymological studies on embryonic and posthatching development of the ovary in the tropical oviparous lizard, *Calotes versicolor*. Journal of Morphology 222:1-10.

Gredler, M. L., T. J. Sanger, and M. J. Cohn. 2015a. Development of the cloaca, hemipenes, and hemiclitores in the Green Anole, *Anolis carolinensis*. Sexual Development 9:21-33.

Gredler, M. L., A. W. Seifert, and M. J. Cohn. 2015b. Morphogenesis and patterning of the phallus and cloaca in the American Alligator, *Alligator mississippiensis*. Sexual Development 9:53-67.

Gregorovicova, M., O. Zahradnicek, A. S. Tucker, P. Velensky, and I. Horacek. 2012. Embryonic development of the monitor lizard, *Varanus indicus*. Amphibia-Reptilia 33:451-468.

Hubert, J. and J. P. Dufaure. 1968. Table de developpement de la vipere aspic: *Vipera aspis*. Bulletin de la Societe Zoologique de France 93:135-148.

Jackson, K. 2002. Post-ovipositional development of the monocled cobra, *Naja kaouthia* (Serpentes: Elapidae). Zoology 105:203-214.

Khannoon, E. R. 2015. Developmental stages of the climbing gecko *Tarentola annularis* with special reference to the claws, pad lamellae, and subdigital setae. Journal of Experimental Zoology Part B: Molecular and Developmental Evolution 324:450-464.

Khannoon, E. R. and S. E. Evans. 2014. The embryonic development of the Egyptian cobra *Naja h. haje* (Squamata: Serpentes: Elapidae). Acta Zoologica 95:472-483.

Khannoon, E. R. and O. Zahradnicek. 2017. Postovipositional development of the sand snake *Psammophis sibilans* (Serpentes: Lamprophiidae) in comparison with other snake species. Acta Zoologica 98:144-153.

Kovtun, M. and H. Sheverdyukova. 2015. Early stages of skull embryogenesis in the Grass snake, *Natrix natrix* (Serpentes, Colubridae). Russian Journal of Developmental Biology 46:222-230.

Lemus, D., J. Illanes, M. Fuenzalida, Y. P. De La Vega, and M. Garcia. 1981. Comparative analysis of the development of the lizard, *Liolaemus tenuis tenuis* . II. A series of normal postlaying stages in embryonic development. Journal of Morphology 169:337-349.

Montero, R., C. Gans, and M. Luisa Lions. 1999. Embryonic development of the skeleton of *Amphisbaena darwini heterozonata* (Squamata: Amphisbaenidae). Journal of Morphology 239:1-25.

Muthukkaruppan, V., P. Kanakambika, V. Manickavel, and K. Veeraraghavan. 1970. Analysis of the development of the lizard, *Calotes versicolor*: A series of normal stages in the embryonic development. Journal of Morphology 130:479-489.

Noro, M., A. Uejima, G. Abe, M. Manabe, and K. Tamura. 2009. Normal developmental stages of the Madagascar ground gecko *Paroedura pictus* with special reference to limb morphogenesis. Developmental Dynamics 238:100-109.

Rapp Py‐Daniel, T., A. Kennedy Soares De‐Lima, F. Campos Lima, A. Pic‐Taylor, O. Rodrigues Pires Junior, and A. Sebben. 2017. A staging table of post‐ovipositional development for the South American Collared Lizard *Tropidurus torquatus* (Squamata: Tropiduridae). Anatomical Record 300:277-290.

Roscito, J. G. and M. T. Rodrigues. 2012. Embryonic development of the fossorial gymnophthalmid lizards *Nothobachia ablephara* and *Calyptommatus sinebrachiatus*. Zoology 115:302-318.

Rosenberg, H. I., A. M. Bauer, and A. P. Russell. 1989. External morphology of the developing hemipenes of the dwarf chameleon, *Bradypodion pumilum* (Reptilia: Chamaeleonidae). Canadian Journal of Zoology 67:884-890.

Zehr, D. R. 1962. Stages in the normal development of the common garter snake, *Thamnophis sirtalis sirtalis*. Copeia 1962:322-329.
